# *Metapolystoma ohlerianum* n. sp. (Monogenea: Polystomatidae) from *Aglyptodactylus madagascariensis* (Anura: Mantellidae)

**DOI:** 10.1007/s11686-023-00668-z

**Published:** 2023-03-17

**Authors:** Willem Landman, Olivier Verneau, Miguel Vences, Louis du Preez

**Affiliations:** 1 Unit for Environmental Sciences and Management, North-West University, Potchefstroom Campus, Private Bag X6001, Potchefstroom, 2520 South Africa; 2 Centre de Formation et de Recherche sur les Environnements Méditerranéens, UMR 5110, University of Perpignan Via Domitia, 66860 Perpignan, France; 3 Centre de Formation et de Recherche sur les Environnements Méditerranéens, UMR 5110, CNRS, 66860 Perpignan, France; 4 Zoological Institute, Braunschweig University of Technology, 38106 Braunschweig, Germany; 5 South African Institute for Aquatic Biodiversity, Private Bag 1015, Makhanda, 6140 South Africa

**Keywords:** *Aglyptodactylus madagascareniensis*, Life cycle, Madagascar, *Metapolystoma*, Polystome

## Abstract

**Purpose:**

Despite Madagascar’s high amphibian diversity of more than 400 species, only a few polystome species are known from the island. The dissection of frogs from museum collections, together with amphibian and parasite surveys conducted in Madagascar led to the discovery of an undescribed polystome infecting *Aglyptodactylus madagascariensis*. The purpose of this study is to formally describe this species.

**Methods:**

Polystomes recovered from *A. madagascariensis* were stained (Acetocarmine) and mounted (Canada balsam) to facilitate morphometrics and taxonomic drawings. Some specimens were fixed in absolute alcohol, a Bayesian tree inferred from the analysis of concatenated 18S, 28S and COI gene sequences was constructed and pairwise distances were calculated. Parasites collected from archived hosts in museums were used for histology and scanning electron microscopy (SEM).

**Results:**

Polystomes recovered from *A. madagascariensis* display characteristics of the genus *Metapolystoma* and morphologically differed from all other known metapolystomes. The Bayesian phylogeny shows that *Metapolystoma* n. sp. ex. *A. madagascariensis* and *M. falcatum* are sister species with high Bayesian posterior probability. Histological and SEM investigations contributed to morphological descriptions.

**Conclusions:**

Morphological examination supported by phylogenetic analysis and genetic divergences revealed distinct differences from all known metapolystome species, supporting the description of a new species. Differences between the life cycles of *Metapolystoma* and *Polystoma* provided additional evidence for the validity of that genus as taxon. Whereas *Polystoma* may display ovoviviparity on rare occasions after incomplete egg expulsion towards the end of the breeding season, *Metapolystoma* displays true ovoviviparity. We emphasize the need for parasite surveys in Madagascar and recommended for museum material to be examined for polystomes to provide supplementary material and localities for further field investigation.

**Graphical Abstract:**

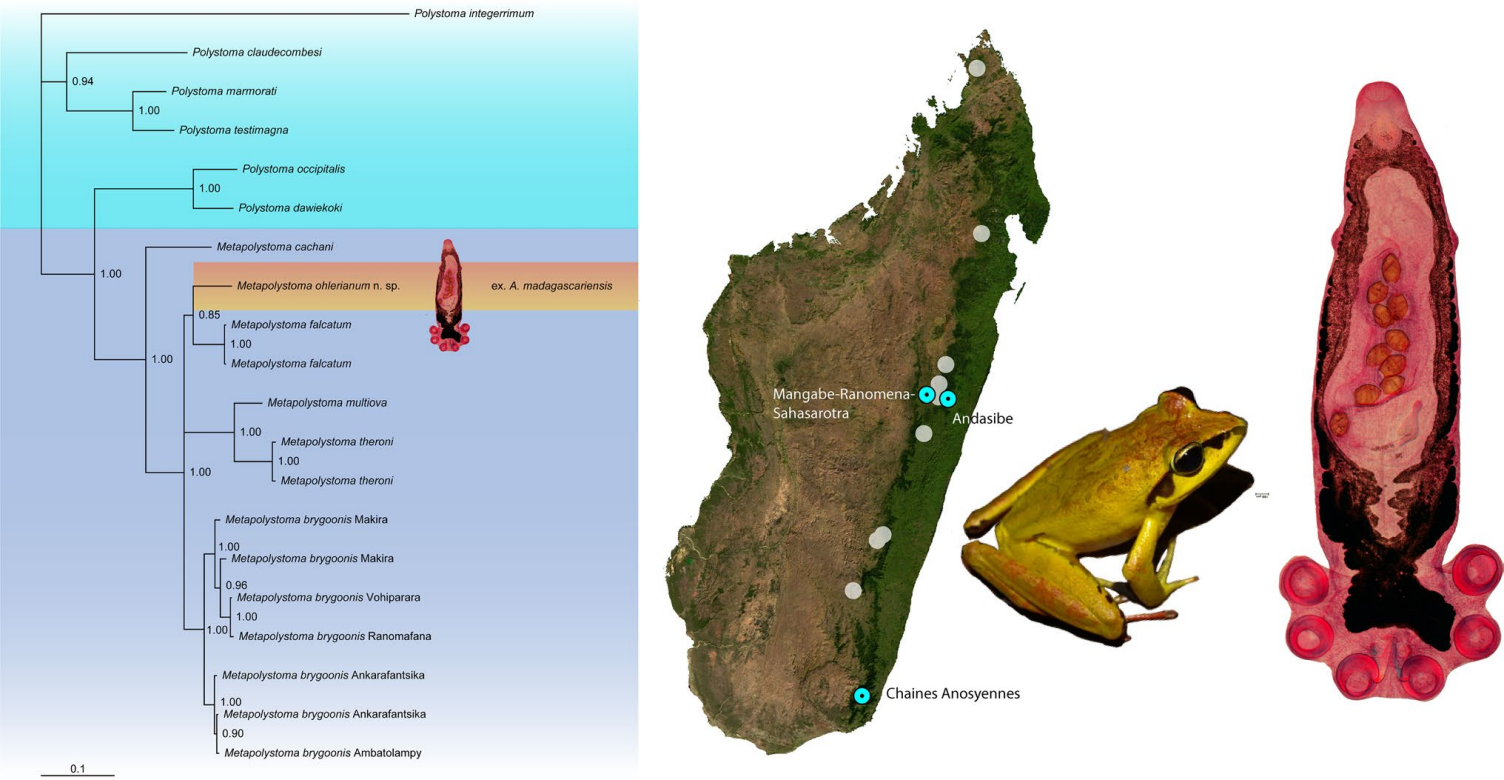

**Supplementary Information:**

The online version contains supplementary material available at 10.1007/s11686-023-00668-z.

## Introduction

Although parasites are often perceived in a negative context, parasitism is the most successful and widespread life-cycle strategy, to the extent that more animals are parasites than not and those that are not parasitic serve as hosts for parasites. This is the result of a long evolutionary history in which hosts and parasites co-evolved and developed successive adaptations [1]. According to [2] parasite diversity correlates strongly with host diversity, which is especially true for amphibian parasites. Since amphibians are exposed to aquatic and terrestrial environments, they can be considered, to some extent, as the perfect host group. Aquatic environments provide ample opportunities for infection. Following completion of metamorphosis, most amphibians move out on land and serve as staple food for all kinds of vertebrates, creating new opportunities for parasite transmission. One example is the African clawed frog *Xenopus laevis* (Daudin 1802) that serves as host for 25 different parasite genera and more than 30 parasite species [3], which, in turn, also illustrate a large diversity of life-cycles.

Monogenea (Polystomatidae) is one of the parasite groups that infects amphibians. Although these parasites were also reported from the Australian lungfish, freshwater chelonians, and the common hippopotamus, they are most abundant in amphibians, more particularly within anurans. It was shown that polystomes would have originated during the evolutionary transition between aquatic and terrestrial vertebrates and subsequently evolved within amphibians [4, 5]. They could have secondarily switched to freshwater turtles and the common hippopotamus from African caecilians [5]. Polystomes co-evolved with their amphibian hosts for at least 250 million years [4], and this may have led to their high diversity of reproductive strategies. All polystomes exhibit direct life cycles which involve a larval oncomiracidium. There are, however, major differences in polystome infestation strategies. Some of these differences are exemplified in the genera *Protopolystoma* infecting a primarily aquatic host, *Metapolystoma* and *Polystoma* infecting amphibious hosts that spend a reasonable time at the water, *Eupolystoma* that infects terrestrial hosts that spend little time at the water and *Pseudodiplorchis* that infects an arid adapted host (see [6, 7]).

Unlike all other polystomes, *Metapolystoma* and *Polystoma* species share two different external reproductive strategies. A neotenic cycle is followed when an oncomiracidium attaches to the gills of a tadpole in pre-metamorphosis. The oncomiracidium then develops into a neotenic parasite and initiates egg production after two to three weeks until the host reaches metamorphosis. Thereafter the parasite detaches from the gills and dies [8–10]. On the contrary, if an oncomiracidium attaches to the gills of a tadpole in pro-metamorphosis, the parasite develops slowly into a urinary bladder destined parasite [11, 12]. This usually takes place at Gosner developmental stage 22 when the hindlegs of the developing tadpole bend at the knee. Upon host metamorphosis, the young parasite leaves the branchial chamber and migrates over the host’s abdomen to the cloaca, where it enters and migrates to the urinary bladder to mature and reproduce in synchrony with the host [11, 12]. However, a third internal reproductive strategy has been documented within *Metapolystoma*. Metapolystomes are partly ovoviviparous where some eggs develop and hatch in utero to reinfect the same host alongside the parent [13]. Eggs that do not hatch in utero hatch after being expelled from the host. The oncomiracidium then infects a tadpole and partakes in either a neotenic branchial or a bladder-destined branchial cycle [13]. Ovoviviparity, though uncommon in *Polystoma*, has been observed in *Polystoma integerrimum* (Frölich 1791) and *Polystoma pelobatis* Euzet and Combes, 1966 [14]. This ovoviviparous cycle is, however, not part of these parasite’s normal reproductive cycle. *Polystoma integerrimum*, similar to *P. pelobatis*, has a short annual egg productive and deposition cycle of only four days per year [6]. One egg may not get expelled by the end of the parasite’s annual reproductive cycle [6, 8] resulting in the retention of an egg. In such an instance, the egg may develop and hatch in utero, whereafter the oncomiracidium will exit the genital atrium and attach alongside the parent [14].

Until the end of the 2020s, *Metapolystoma* (Monogenea: Polystomatidae) was only known from *Ptychadena* Boulenger, 1917 found in Madagascar and the African continent. This genus included *Metapolystoma brygoonis* (Euzet and Combes 1964) from *Ptychadena mascareniens* (Duméril and Bibron 1841) in Madagascar, *Metapolystoma cachani* (Gallien 1956) from *Ptychadena longirostris* (Peters, 1870) in West-Africa and *Metapolystoma porosissimae* Du Preez and Kok 1992 from *Ptychadena porosissima* (Steindachner 1867) in South Africa. It is only recently that Landman *et al*. [15] described, from Madagascar, *Metapolystoma ansuanum* Landman, Verneau, Raharivololoniainan and Du Preez 2021 from *Boophis luteus* (Boulenger 1882), *Metapolystoma falcatum* Landman, Verneau, Raharivololoniainan and Du Preez 2021 from *Boophis doulioti* (Angel 1934), *Metapolystoma multiova* Landman, Verneau, Raharivololoniainan and Du Preez 2021 from *Boophis occidentalis* Glaw and Vences, 1994, *Metapolystoma theroni* Landman, Verneau, Raharivololoniainan and Du Preez 2021 from *Boophis madagascariensis* (Peters 1874) and *Metapolystoma vencesi* Landman, Verneau, Raharivololoniainan and Du Preez 2021 from *Boophis albilabris* (Boulenger 1888). After the examination of archived collections of Malagasy frogs in Antanarivo (Madagascar) and Paris (France) together with field surveys in Madagascar, *Aglyptodactylus madagascariensis* (Duméril 1853) was found to be infected with a polystomatid flatworm. Based on molecular evidence [16], this parasite represents a yet undescribed *Metapolystoma* species. Our objectives were therefore to formally describe this new species recovered from *A. madagascariensis* and to indicate the value of museum collections in the assessment of parasite diversity, especially in protected areas where the collection of potential hosts is restricted.

## Material and Methods

### Host and Parasite Sampling

**Fig. 1:**
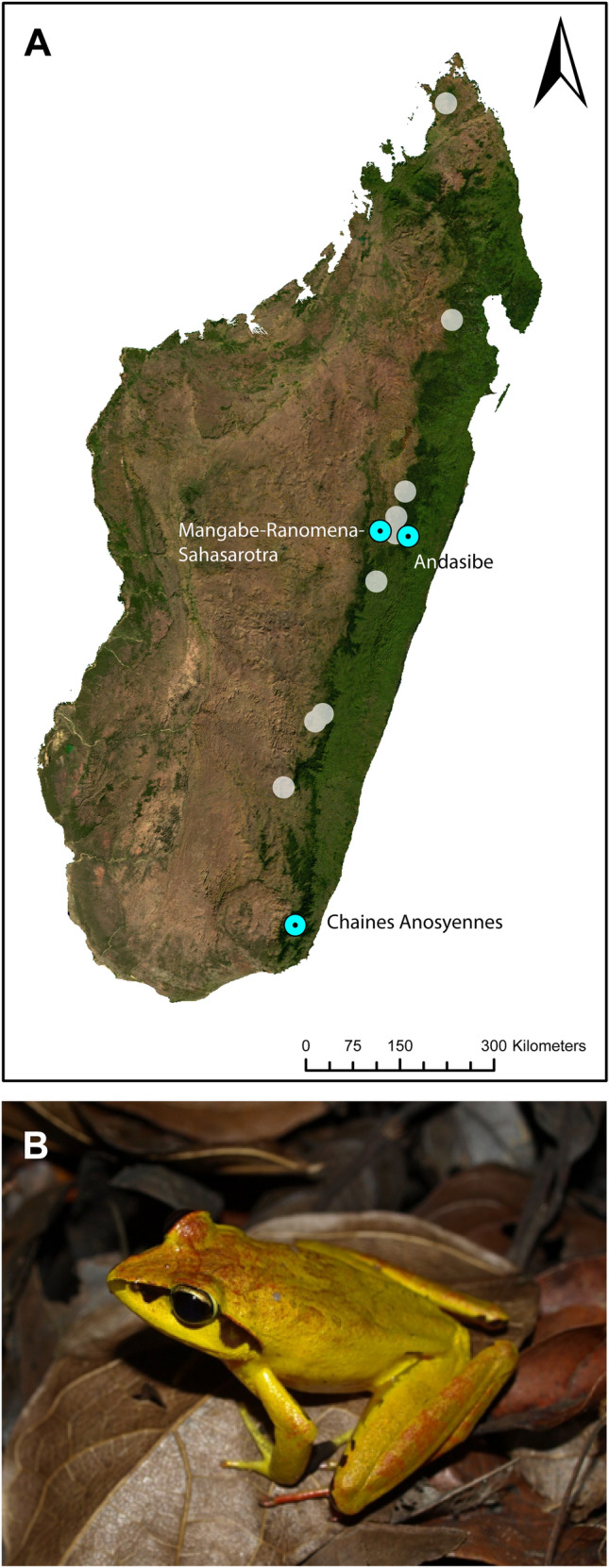
**A** Distribution of *Aglyptodactylus madagascareniensis* in Madagascar with sites where parasites were found. **B** *Aglyptodactylus madagascariensis.* ArcMap 10.5.1 (Esri, California) was used to compile a host distribution map, based on genetically verified records of Köhler et al. [17]

In January 2005, two specimens of *A. madagascariensis,* collected at Andasibe, were examined and found to be uninfected with polystomes. In February 2006, a variety of formalin preserved specimens were examined at the Tsimbazaza Zoological Garden, Antananarivo, Madagascar for polystomes. Among them, 12 specimens of *A. madagascariensis* collected in November 2005 from two distinct localities of Madagascar, namely Besariaka and Mangabe-Ranomena-Sahasarotra (Fig. 1A) were examined. In May 2006, a variety of Malagasy frog species were examined at the Muséum National d’Histoire Naturelle of Paris. A small cut was made across the lower abdomen to expose the urinary bladder which was pulled out for inspection. Thirty-three specimens of *A. madagascariensis* were examined. These specimens were collected in November 1971 at Chaines Anosyennes, Madagascar (Fig. 1A) Host specimens from Chaines Anosyennes were collected outside the currently known distribution of *A. madagascariensis* (Fig. 1A) and may be *Aglyptodactylus australis* Köhler, Glaw, Pabijan, and Vences, 2015. For this reason, specimens from this locality were not included in the morphometric  description. In February 2008 following a rainstorm at Andasibe (Fig. 1A), Madagascar, two specimens of *A. madagascariensis* (Fig. 1B) were collected dead on the road and 32 frogs were collected by hand around various forest pools in the area. Frogs were euthanized using Ethyl-3-aminobenzoate methanesulfonate (MS222) and dissected to inspect for polystomes. The accessory bladders, Wolffian ducts and kidneys were also examined. Parasites that were retrieved were transferred to glass cavity blocks containing 0.6% amphibian saline solution.

Adult specimens used for morphological examination were fixed and preserved in 10% neutral buffered formalin (NBF) under coverslip pressure. Following staining in a weak acetocarmine solution, they were dehydrated and mounted in Canada balsam. Juvenile specimens were mounted as temporary preparations in ammonium picrate glycerine for examination of sclerites. One specimen for molecular analysis was preserved in 100% ethanol and processed in Verneau *et al*. [16].

### Phylogenetic and Distance Analyses

**Fig. 2:**
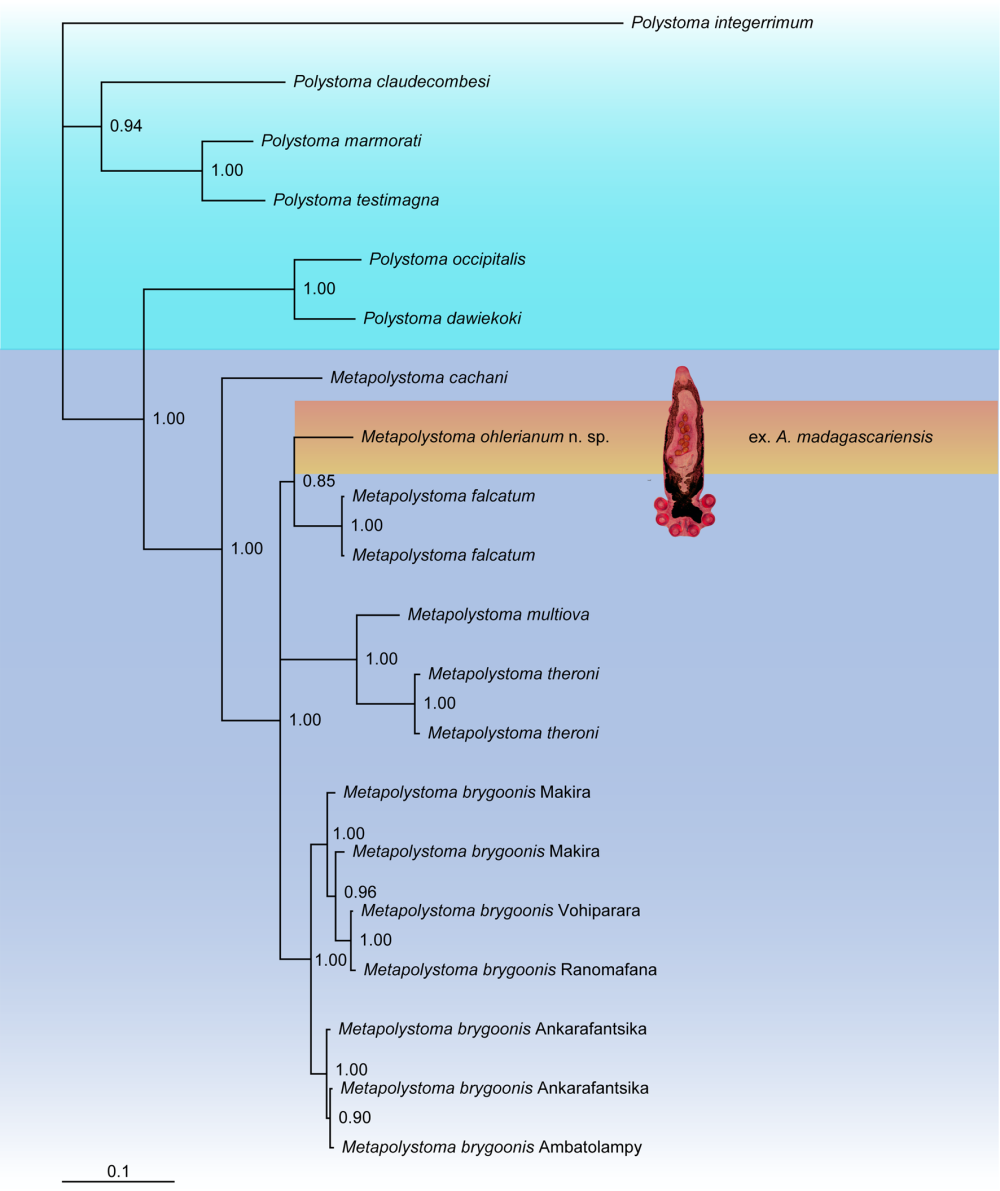
Bayesian tree inferred from the analysis of concatenated 18S, 28S and COI gene sequences. Node values indicate Bayesian posterior probabilities

All 18S, 28S and COI sequences used in this study, except for *Metapolystoma* sp. ex. *A. madagascariensis,* were extracted from Genbank and listed in Landman *et al*. [15] under Accession numbers AM051069, AM051071, AM051073, AM051075, AM157194, AM157204, AM157206, AM157208, AM157217, AM913856, AM913859, AM913860, JF699306, JN800281, JN800283, JN800285–JN800289, JN800291, JN800293, JN800294, FM897262–FM897264, FM897267–FM897270, FM897280, FM897281, FM897284–FM897287, FM897298, FM897300, FM897301, MW054236–MW054249, MW053457 and MW053458 for the description of *Boophis* metapolystomes. Sequences for *Metapolystoma* sp. were also extracted from Genbank, however listed in Verneau *et al*. [16] under Accession numbers FM897266, FM897283 and FM897299 for a global phylogenetic analysis of Madagascan polystomes. Clustal W, which is implemented in Mega 7 [18], was selected to edit and align 18S, 28S and COI sequences independently using default parameters [19]. Resulting 18S and 28S alignments comprised 20 taxa, containing 14 *Metapolystoma* and six distinct *Polystoma* species, with *Polystoma integerrimum* (Frölich 1791) used as an outgroup, while the resulting COI alignment comprised only 18 taxa. The 18S, 28S and COI sequences were finally concatenated in a single alignment for Bayesian analysis. 18S and 28S sequences were treated as two separate partitions while COI sequences were treated as three distinct partitions according to their codon position. A two substitution rates model was selected for the 18S partition and a GTR + I model for the 28S partition following the Akaike Information Criterion (AIC) implemented in Modeltest 3.06 [20]. Six types of substitutions and four gamma rates categories were applied for COI partitions one and two, while six types of substitutions with invariable-gamma rates were applied for COI partition three. Evolutionary parameters for the five partitions were estimated separately with MrBayes 3.04b [21]. Four chains were run for ten million generations, sampling every 100 cycles. The first 10 000 trees (10%) were removed at the burn-in phase. The Bayesian consensus tree was drawn (Fig. 2) and inspected with Tree View version 1.6 [22]. Corrected pairwise distances used for species delimitation were calculated independently for the three genes in MEGA version 7 with the Kimura 2-parameter model [23]. Total character differences between species were also estimated for all three genes.

### Morphology and Morphometry

A Zeiss Imager Axio10 compound microscope (Zeiss, Germany), fitted with a Zeiss Axio cam 305 camera (Zeiss, Germany) was used to investigate and photograph parasites. Taxonomic measurements in micrometres were captured using the Zeiss Zen Blue elements (Zeiss, Germany) software program. A Nikon AZ100M stereomicroscope (Nikon, Netherlands) was used to capture full-body micrographs of type specimens. Adobe Illustrator CC (Adobe, California) was used to prepare illustrations from micrographs.

Eggs were measured in length (x) and width (y), plotted on a scatterplot, and encircled by a 95% confidence ellipse with the mean $\left( {\overline{{\text{x}}} ;\overline{{\text{y}}} } \right)$ as pivot in order to discriminate between different species groups. Measurements came from the type series of *M. ansuanum*, *M. falcatum*, *M. multiova*, *M. porosissimae*, *M. theroni* and *M. vencesi* and from Kulo [24] for *M. cachani*. Concerning *M. brygoonis*, measurements were from parasites collected in September 1988 at Ranomafana, Madagascar that were stored in the polystome collection of the North-West University, Potchefstroom, South Africa. Marginal hooklets were measured according to the protocol of Du Preez and Maritz [25] in order to discriminate species groups.

### Histology

Specimens retrieved from archived host material were not fixed flat and thus not suitable as whole mounts. They were preserved in 10% neutral buffered formalin (NBF) for histological examination. Specimens were washed and dehydrated in an ethanol series, impregnated with paraffin wax and embedded for sectioning using a Slee MPS histocene embedding machine (Slee, London, UK). Specimens were sectioned at 6 μm using a Reichert Jung motorised microtome (Reichert-Jung, Nosloch, Germany). Sections were stained with routine eosin and Harris’ hematoxylin and mounted using Entellan mounting medium [26].

### Sclerite Isolation and Scanning Electron Microscopy

Sclerotized hooks were isolated from adult parasites through enzyme digestion of soft body parts using Proteinase K [200 µg/ml] (see [27]). Haptors were separated and cut into smaller fragments, rehydrated in distilled water, and placed on a Millipore filter (pore size 0.5 μm). Specimens were then covered with the enzyme solution and incubated at 50 ℃ for 10 to 15 min. Subsequently, distilled water was forced through the filter with a syringe in order to rehydrate specimens and to remove excess salts and debris. Digestion and rehydration steps were repeated seven to eight times until all soft tissue was removed from the hooks. Filter papers with sclerite hooks were dried overnight in a desiccator prior to the SEM analysis. Dried material was mounted on aluminium SEM stubs with carbon tape and sputter-coated for 90 s with gold palladium using a SPI-Module Sputter Coater (Spi Supplies: Westchester, PA, USA). SEM images were captured using a Phenom Pro Desktop SEM (Phenom-World BV., Eindhoven, Netherlands).

## Results

### Levels of Infection

Two of the seven frogs examined from Mangabe-Ranomena-Sahasarotra were infected. One specimen was infected with one mature and eight juvenile parasites and the second with 15 mature parasites. All five frogs from Besariaka were uninfected. Of the 33 frogs from Chaines Anosyennes that were examined at the Muséum National d’Histoire Naturelle of Paris, a single frog was infected with nine mature parasites. Finally, four of the 34 live frogs from the Andasibe region were infected with a total of four mature and 17 juvenile parasites (prevalence 11.8%, mean intensity 5.3). All four frogs were infected with small, subadult and mature parasites.

### Phylogenetic Relationships and Genetic Divergences Within Metapolystomes

The Bayesian phylogeny shows that *Metapolystoma* n. sp. ex. *A. madagascariensis* and *M. falcatum* are sister species with high Bayesian posterior probability (Fig. 2). The divergence estimates with standard deviations and total differences between *Metapolystoma* n. sp. and *M. falcatum* are 0.052% ± 0.00 and 1 for the 18S, 0.141% ± 0.001 and 2 for the 28S and 9.372% ± 0,018 and 28 for the COI. These values exceed the species level threshold of 0.07% for 28S and 2% for COI as proposed by du Preez *et al*. [18], suggesting that it is a new species.

### *Metapolystoma ohlerianum* n. sp. (Fig. 3)

**Fig. 3:**
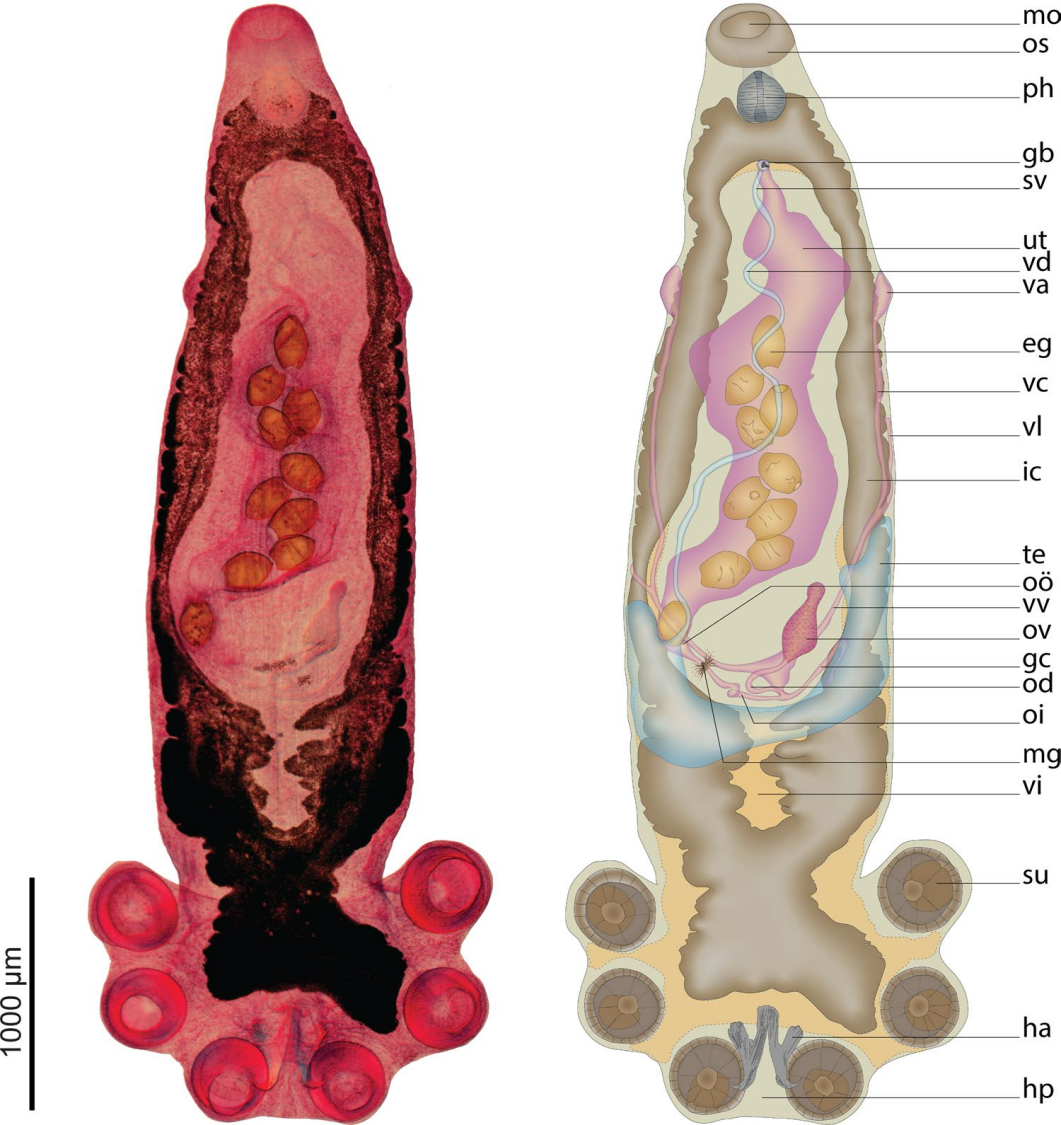
Ventral view of *Metapolystoma ohlerianum* n. sp. holotype. *eg* egg, *gb* genital bulb, *gc* genito-intestinal canal, *ha* hamuli, *hp* haptor, *ic* intestinal caecum, *mo* mouth, *mg* Mehlis gland, *od* oviduct, *oi* oö-vitelline canal, *oö* oötype, *os* false oral sucker, *ov* ovarium, *ph* pharynx, *su* sucker, *sv* semen vesicle, *te* testis, *ut* uterus, *va* vagina, *vc* vaginal cannel, *vd* vas deferens, *vi* general position of vitelline (not visible on type), *vl* vitelline duct, *vv* vitello-vaginal cannel

#### Taxonomic Summary

Class Monogenea van Beneden, 1858

Order Polystomatidea Lebedev, 1988

Family Polystomatidae Gamble, 1896

Genus *Metapolystoma* Combes, 1976

#### Type Host

*Aglyptodactylus madagascariensis* (Duméril 1853), Mantellidae (Fig. 1B).

#### Site in Host

Adults in urinary bladder, some juveniles in the Muller ducts and urinary bladder.

#### Type Material

Morphological descriptions based on 13 mature and 2 juvenile parasites. Holotype NMBP 583 (Fig. 3) and Paratypes NMBP 584–586 deposited in the Parasitic Worm collection, National Museum, Aliwal Street, Bloemfontein 9301, South Africa.

#### Voucher Material

Remaining specimens in parasite collection at North-West University, Potchefstroom, South Africa.

#### Type Locality

Holotype NMBP 583 and Paratypes NMBP 585–586 from Andasibe, Madagascar (− 18.938753S; 48.414471E). Paratype NMBP 584 from Mangabe-Ranomena-Sahasarotra, Madagascar.

#### Zoobank Registration

The Life Science Identifier (LSID) of the article is: urn:lsid:zoobank.org:pub:564F7CC4-4EA4-45BD-A480-9CB98304D0F2. The life science identifier (LSID) of the new name *Metapolystoma ohlerianum* n. sp. Landman *et al*. is: urn:lsid:zoobank.org:act:9B1ECEB9-F5B6-4C46-9199-30035F5F6DE8.

#### Etymology

In recognition of Dr. Annemarie Ohler, Muséum National d’Histoire Naturelle of Paris, for her support over years and for allowing the examination of museum materials.

### Description

**Fig. 4:**
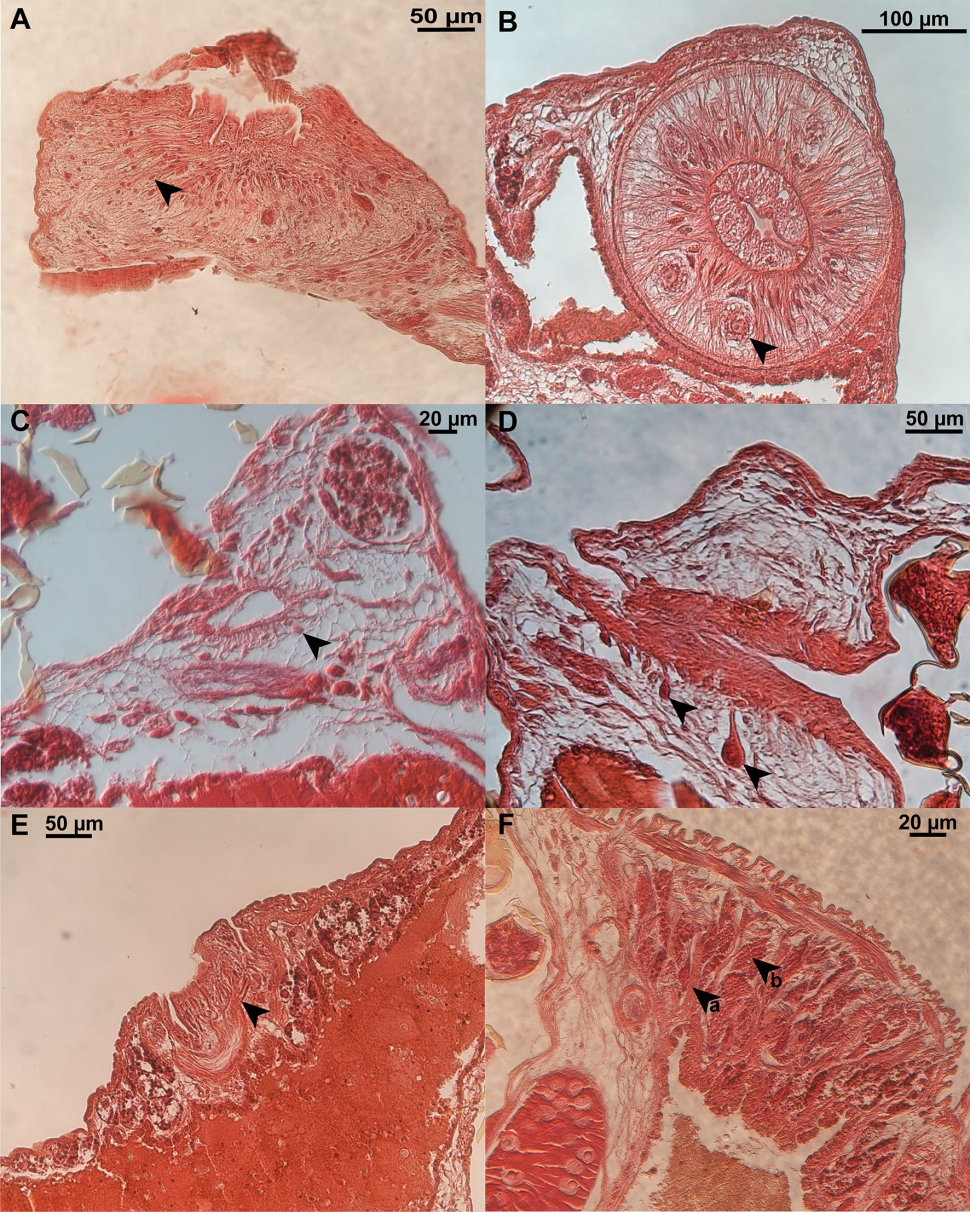
Compound microscope micrograph of histology sections through *Metapolystoma ohlerianum* n. sp. **A** Section through false oral sucker interspersed with mucous gland cells (arrow). **B** Cross-section through pharynx imbedded with glandular cells (arrow). **C** Section through Mehlis gland (arrow).  **D** Section through genital bulb surrounded with glandular cells (arrows). **E** Section through vagina with the vaginal canal branching out (arrow) to form multiple vaginal pores.  **F** Section through testis showing testis follicles (arrow b) surrounded with connective tissue (arrow a)

Measurements were obtained from 13 mature and two juvenile bladder parasites and are given in micrometres. Body pyriform (Fig. 3), dorsoventrally flat, 3508–5042 (4517 ± 700; 4) long, 1209–1758 (1515 ± 228; 4) wide, with widest section 53–69% (58% ± 7.2; 4) of total length measured from anterior end, body length 2.2–4.2 (3.1 ± 0.9; 4) times greater than width. Width at vagina 1041–1340 (1210 ± 149; 4). Mouth 228–284 (250 ± 26; 4) in diameter, sub-ventral surrounded by false oral sucker. False oral sucker interspersed with mucous gland cells (Fig. 4A). Medial pharynx length 251–297 (282 ± 21; 4) greater than width 226–284 (257 ± 27; 4), imbedded with glandular cells throughout its length (Fig. 4B). Intestine bifurcates at 14–17% (16% ± 1; 4) of total length from most anterior point, converging posteriorly at 58–74% (69% ± 8; 4) of total length, from most anterior point, extending into haptor; no prehaptoral anastomoses. Lateral diverticula length equal to width. Medial diverticula posterior to ovary, length greater than with.

Ovary 349–427 (378 ± 43; 3) long, 136–190 (169 ± 28; 3) wide, elongate, not lobed, positioned posterior to midbody, 7–9% (8% ± 1; 3) of body length, 2–3 (2 ± 0.3; 3) times longer than wide. Oviduct 422–491 (455 ± 35; 3) long, 18–49 (34 ± 9; 3) wide. Mehlis’ glands large, surrounding the base of the oötype (Fig. 4C). Uterus large, occupying one quarter of body proper, sack like, surrounded by accessory glands at genital bulb connection (Fig. 4D), containing up to 61 ovoid, operculate eggs 187–242 (211 ± 12.6; 43) long, 104–182 (127 ± 21.3; 49) wide, some contain fully developed oncomiracidia; some hatched intrauterine oncomiracidia present. Two parallel vaginae 195–270 (229 ± 27; 7) long, 93–177 (137 ± 33; 7) wide, on lateral margins, bearing multiple marginal openings formed by branching vaginal canal (Fig. 4E), vaginal vestibule cup shaped, 24–27% (25% ± 1.2; 4) from anterior. Vitellaria extended throughout most of body, stretching in between haptoral suckers, surrounding the female reproductive organs. Genito-intestinal cannel prominent 469–706 long, 22–51 (33 ± 9; 2) wide, situated posterior to ovary.

**Fig. 5:**
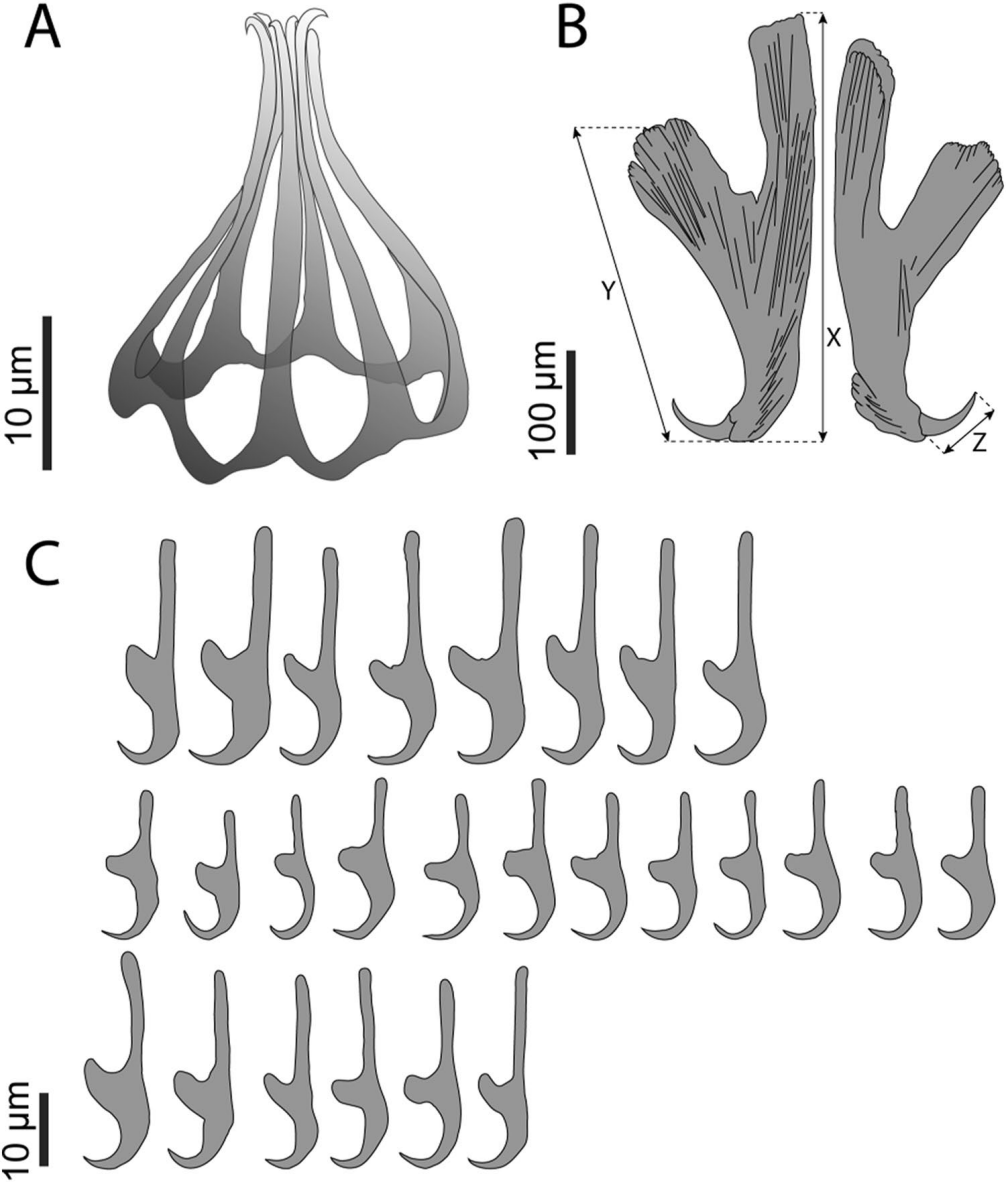
*Metapolystoma ohlerianum* n. sp. **A **Genital crown from holotype **B** Hamuli from holotype, X - hamulus handle length, Y - hamulus guard length, Z - hamulus hook length **C** Marginal hooklets 1 (top), 2–7 (middle) and 8 (bottom) from holotype and paratypes

Testis follicular, encapsulated in connective tissue that forms a tube (Fig. 4F), U-shaped, mainly positioned posterior to the ovary with two lateral processes extending forward along the lateral line past the ovary to a position about 25% from anterior end of body proper, ventral to intestine. Lateral fields of testis pushed into posterior position when the uterus is filled with eggs, sometimes causing the testis to be asymmetrical. Vas deferens widens anteriorly to form sinuous semen vesicle 14–33 (22 ± 2; 3) wide, 38–88 (62 ± 25; 3) long, narrowing towards genital bulb, opening in common genital opening. Genital pore opening ventral, directly posterior to intestinal ceca bifurcation, 5–15% (12% ± 5; 4) of total length from most anterior point, genital bulb muscular 59–72 (66 ± 4; 7) in diameter, surrounded by glandular cells, armed with a genital crown 21.6–27.4 (23.5 ± 2; 6) in diameter, with seven genital spines 26.7–32.0 (29.7 ± 1.7; 17) long (Fig. 5A).

**Fig. 6:**
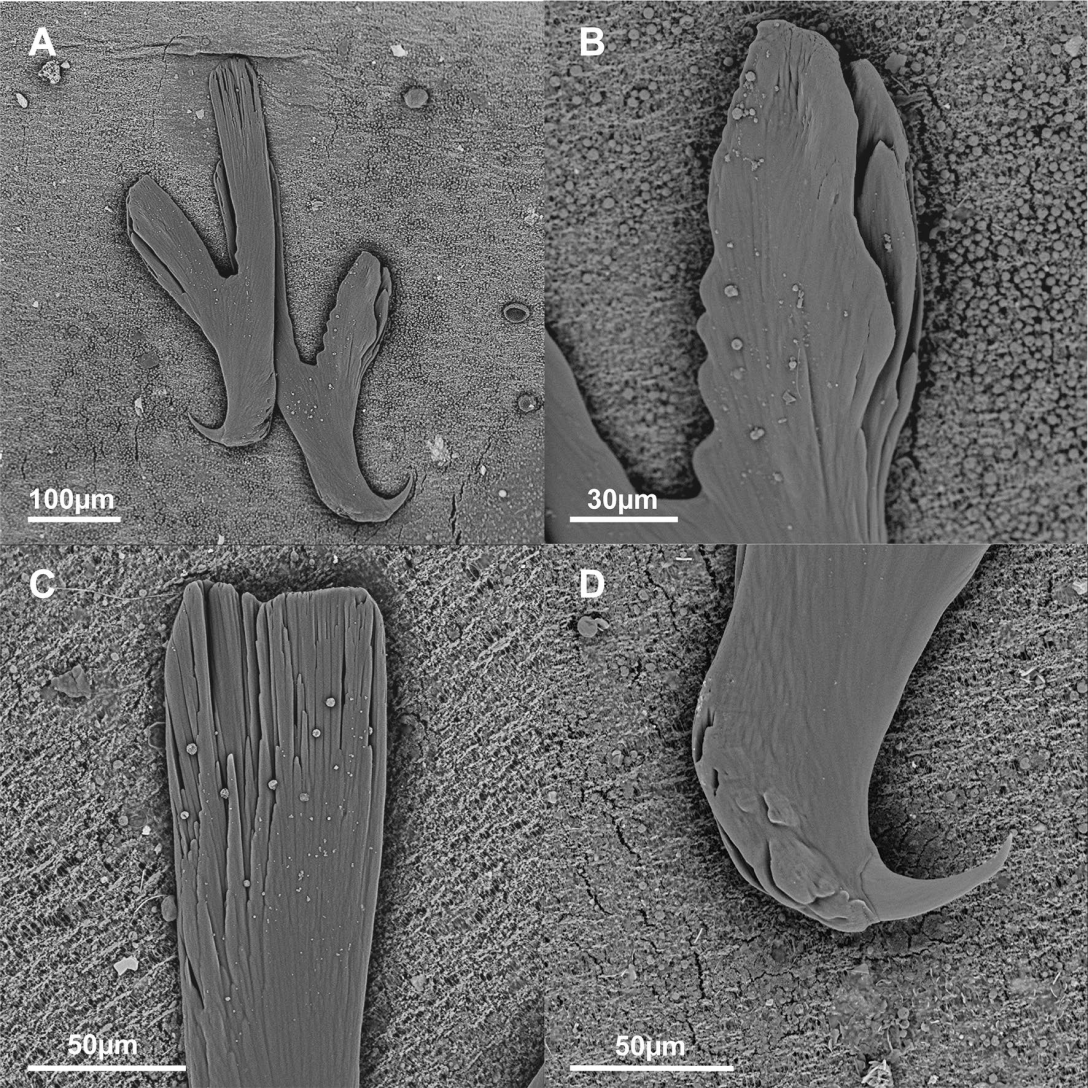
Scanning electron micrographs of *Metapolystoma ohlerianum* n. sp. showing **A** Hamuli. **B** Deep grooves in hamulus guard for muscle attachment. **C** Deep grooves in hamulus handle for muscle attachment. **D** Deep grooves in hamulus head for muscle attachment

**Fig. 7:**
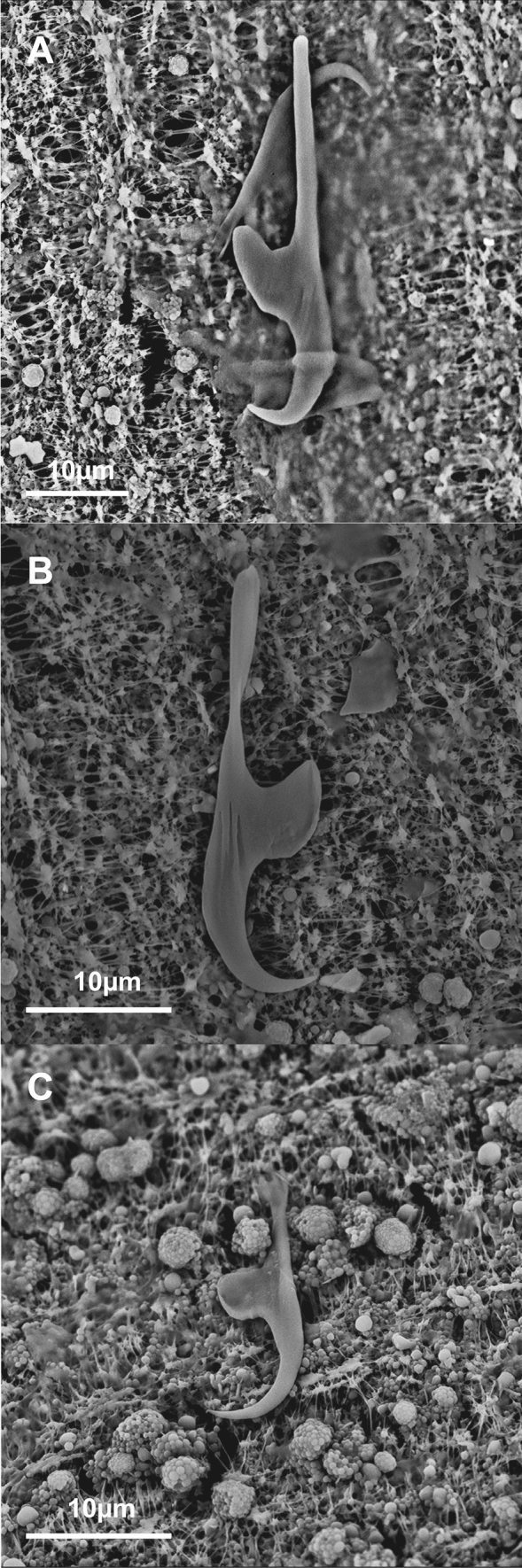
Scanning electron micrographs of *Metapolystoma ohlerianum* n. sp. showing **A** Lateral grooves on marginal hooklet one for muscle attachment. **B** Lateral grooves on marginal hooklet eight for muscle attachment. **C** The absence of lateral grooves for muscle attachment on marginal hooklets two to seven

Posterior haptor 1208–1280 (1239 ± 32; 4) long, 1647–2055 (1852 ± 168; 4) wide, 24–34% (28% ± 4.6; 4) of body length, bearing three pairs of cup-shaped haptoral suckers equal in diameter 296–526 (387 ± 51; 27). Well-developed hamuli between posterior–most haptoral suckers with deep cut between handle and guard (Fig. 5B), handle length X 340–475 (391 ± 42; 14), guard length Y 263–392 (315 ± 27; 15), and hook length Z 57–69 (63 ± 3.6; 21), some hamuli with posterior crest (Fig. 5B right). Marginal hooklets placed as for other polystomes: pairs one and two between hamuli, marginal hooklet pairs three to five embedded in suckers, pairs six to eight between anterior suckers. Marginal hooklet pairs one 29.6–33.6 (31.7 ± 1.3; 8) long (Fig. 5C top) and eight 25.9–30.0 (27.5 ± 1.6; 8) long (Fig. 5C bottom), larger than pairs two to seven 17.8–22.2 (20.6 ± 1.2; 12) long (Fig. 5C middle). Hamulus guard, handle and head deeply grooved for muscle attachment (Fig. 6A–D). Marginal hooklet pairs one (Fig. 7A) and eight (Fig. 7B) laterally grooved between hook tip and guard for muscle attachment, hooks two to seven not grooved (Fig. 7C).

### Egg and Marginal Hooklet Morphometrics

**Fig. 8:**
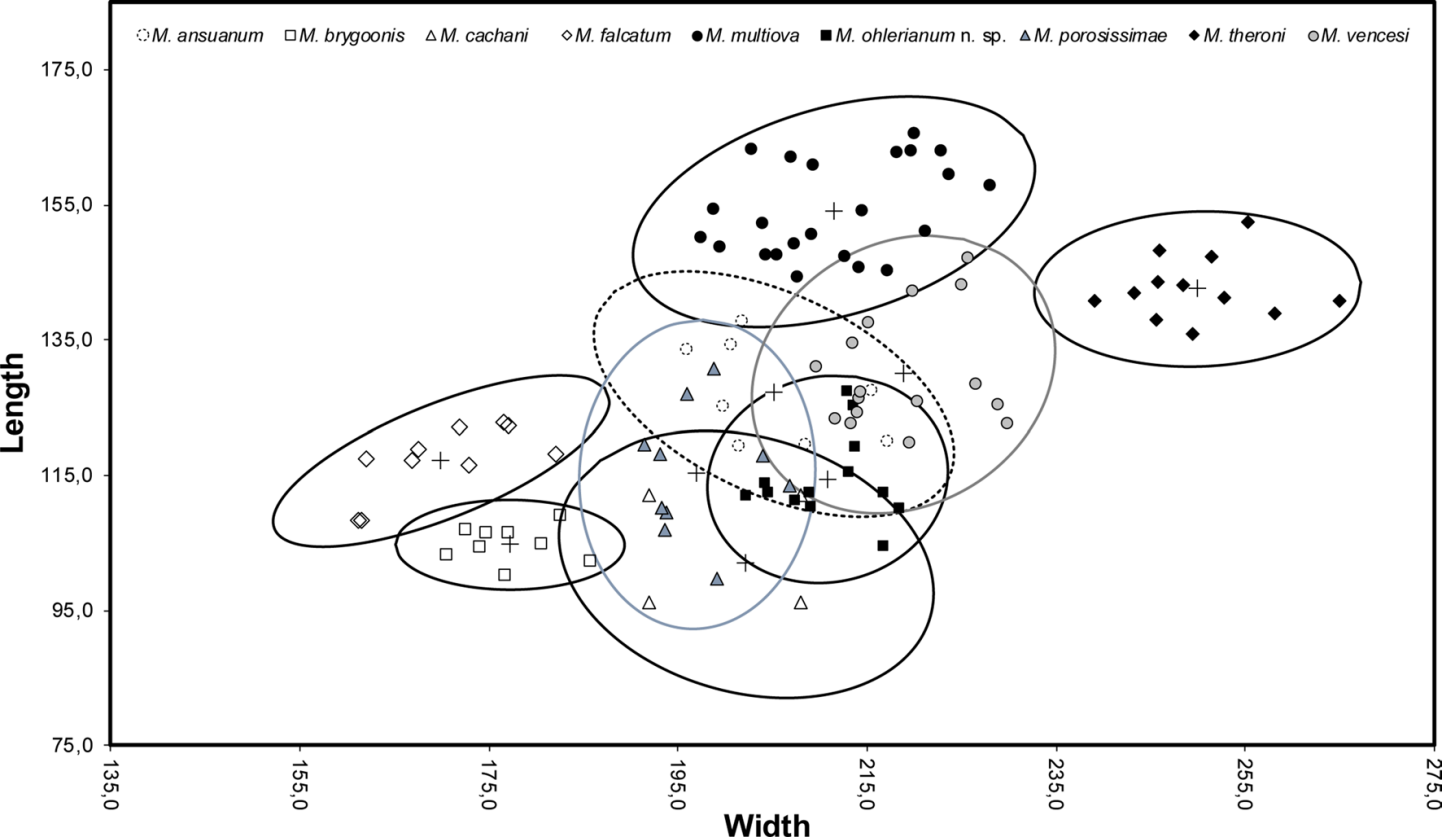
Scatter diagram of egg length plotted against egg width for all known *Metapolystoma* spp. including *Metapolystoma ohlerianum* n. sp. The ellipses represent 95% of the confidence interval about the mean

Egg morphometric measurements separated *M. ohlerianum* n. sp. from *M. brygoonis*, *M. falcatum*, *M. multiova* and *M. theroni* with no overlap in the scatterplot (Fig. 8). However, they did not separate *M. ohlerianum* n. sp. from *M. ansuanum*, *M. cachani*, *M. porosissimae* and *M. vencesi.*

**Fig. 9:**
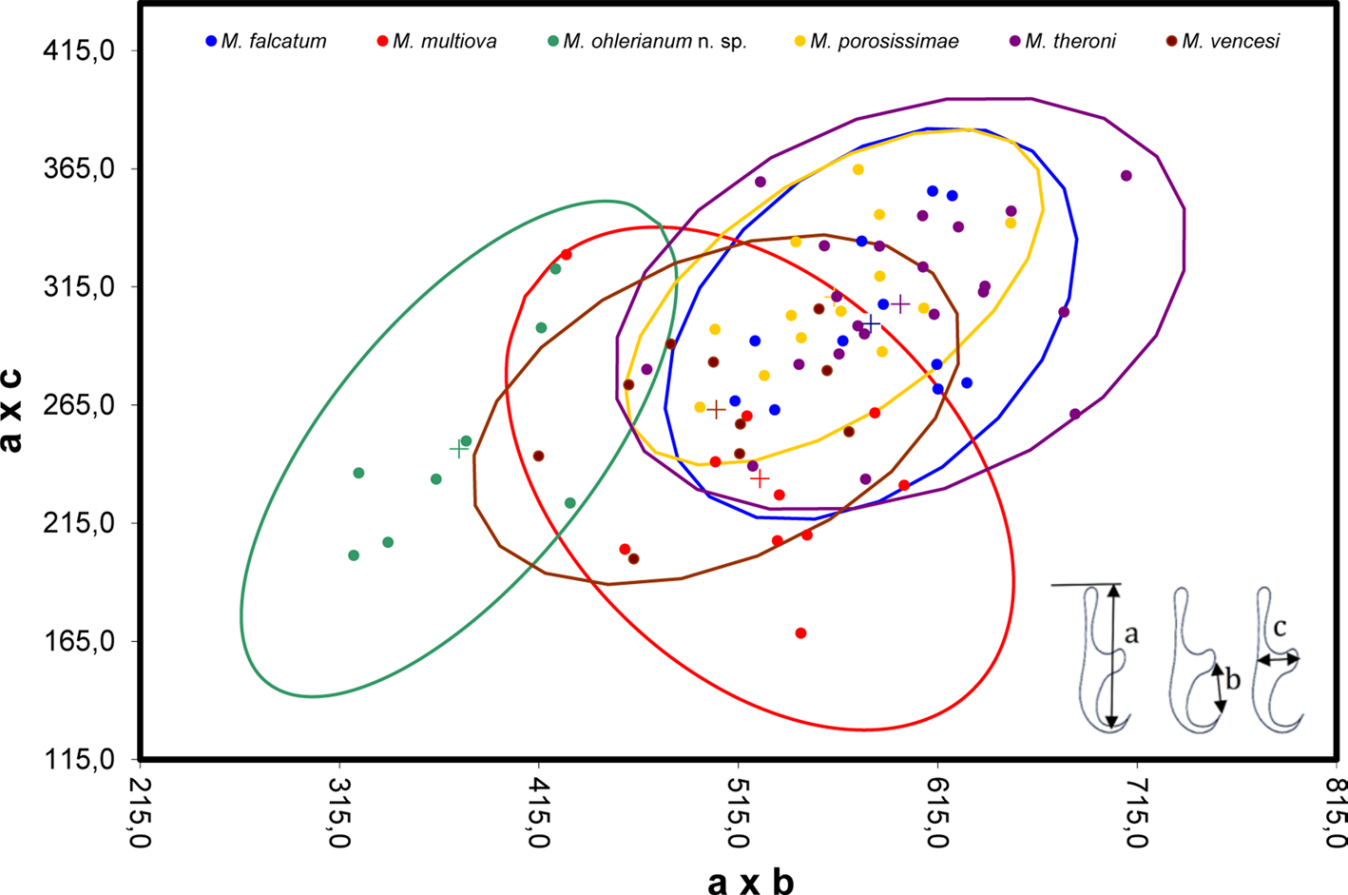
Scatter diagram of a × c plotted against b × c for *Metapolystoma* spp., for which measurements could be obtained. The ellipses represent 95% of the confidence interval about the mean

Marginal hooklet morphometric measurements separated *M. ohlerianum* n. sp. from *M. falcatum* with no overlap in the scatterplot (Fig. 9). Marginal hooklet morphometric measurements, however, did not separate *M. ohlerianum* n. sp. from *M. multiova*, *M. porosissimae*, *M. theroni* and *M. vencesi*.

### Remarks

Whereas all metapolystomes described to date have tubiform uteri that convolute between the oötype and the genital pore, *Metapolystoma ohlerianum* n. sp. differs from all *Metapolystoma* species in that it has a sack-like uterus. Furthermore, *M. ohlerianum* n. sp., which has seven genital spines, differs from all other metapolystomes except *M. theroni*. However, these two species differ from each other from egg morphometrics and hamulus hook length.

## Discussion

With *M. ohlerianum* n. sp., there are now seven *Metapolystoma* species known from Madagascar. It can, however, be expected that more *Metapolystoma* species await discovery from *Aglyptodactylus* species. *Aglyptodactylus madagascariensis* is a predominant terrestrial species that occurs in the eastern forests of Madagascar and spends most of its time between leaf litter during the daytime, and in and around temporary pools at night [28, 29]. These frogs are explosive breeders that breed following heavy rains [29, 30]. Since these frogs gather in great numbers to breed, many frog and parasite eggs are deposited in relatively small, temporary pools. These conditions are ideal for parasite reinfection since many tadpoles are concentrated in small waterbodies, which increases the probability of a single oncomiracidium to find a host. Although the nested phylogenetic position of *Metapolystoma* within *Polystoma* raises questions about its validity as a genus (see [31]), both phylogenetic and morphological results suggest that *Metapolystoma* is a distinct clade that we recommend to be kept as a separate taxon for the time being (see also [15]). *Metapolystoma,* as with *Polystoma*, has both mature branchial and bladder adult forms. However, metapolystomes present true ovoviviparity where eggs develop and hatch prior to egg deposition, to reinfect the same host [32]. This is also evident in re-examined material of Landman *et al*. [15], within *Boophis madagascariensis,* i.e., the host of *M. theroni*, which was infected with 23 recently established oncomiracidia, 19 subadult (non-reproductive) and one mature (reproductive) parasite. The same phenomenon has been observed for *B. doulioti*, i.e., the host of *M. falcatum*, which was infected with five small parasites in the Müller ducts and respectively six subadult and one mature parasite in the urinary bladder (re-examined material of Landman *et al*. [15]), suggesting multiple reinfection events. Some *Polystoma* species present ovoviviparity, however, this cannot be considered as part of the parasite’s natural reproductive cycle. This difference between the life-cycles of the two genera thus provides further evidence supporting *Metapolystoma* as a distinct group.

Although great emphasis has been placed on Madagascar as a biodiversity hotspot [33], its parasite diversity remains underexplored, including that of amphibian polystomes. This is especially true when taking into consideration Madagascar’s rich amphibian diversity of over 400 species [34] of which 83 species were investigated for polystomes at a few selected localities [16]. These studies led to the description of two new polystome genera within amphibians [35, 36] and 11 new polystome species [15, 35–38], knowing that undescribed *Madapolystoma* species still await description [38]. Furthermore, *Metapolystoma brygoonis, *which could be a complex of species [15], given the high genetic variation observed in its host in Madagascar [39], and therefore requires further investigation. At the current rate of species extinction many species are becoming extinct before they are formally described [40–42]. The biodiversity of Madagascar is particularly threatened as a result of forest fragmentation and habitat loss [43, 44]. The hosts of some undescribed Malagasy polystome species that were discovered during our studies are now Endangered. In fact, 80 (26%) of the 311 anurans assessed in Madagascar are listed as being Endangered and 21 (7%) as Critically Endangered [45]. Consequently, due to collecting permit restrictions, the parasite diversity of these frogs will probably never be assessed. Extinction of any species has a ripple effect as each species is a package of the host with its species-specific parasites. With amphibians being the most threatened vertebrates and their numbers continuously declining [45], this statement is particularly true for their polystomatid parasites as well. Especially since amphibian polystomatids are generally considered to be host specific [31, 37, 47–50]. With parasites forming such an intricate part of biodiversity and ecology, the in-depth study of polystomes has the added advantage that in the process we learn more about the host itself. This is especially true when taking into consideration that the phylogeny of polystomes provides insights into the biogeographical origin of their amphibian hosts [16, 37] and ultimately aid in their conservation [37]. Madagascar is globally ranked twelfth with regard to amphibian richness [51], which suggests that many more Malagasy polystome species are still undiscovered and undescribed. We therefore emphasize the need for further parasite surveys and recommend that, as with this study, museum collections should be examined for parasites. The investigation of museum specimens will thus provide localities for further field studies, which will help in finding supplementary material for molecular analyses and morphological descriptions.

## Supplementary Information

Below is the link to the electronic supplementary material.

Supplementary file1 (PDF 182 KB)

## Data Availability

Raw data generated from this study is available upon personal request from the corresponding author.
