# Supplementary material for: Metapolystoma ohlerianum n. sp. (Monogenea: Polystomatidae) from Aglyptodactylus madagascariensis (Anura: Mantellidae)
Source: Acta Parasitol. 2023 Mar 17;68(2):344–58. doi: 10.1007/s11686-023-00668-z (PMC10281909; doi:10.1007/s11686-023-00668-z)
Supplement: Supplementary file 1 — Supplementary file1 (PDF 182 KB) [file 11686_2023_668_MOESM1_ESM.pdf]

NWU®
NORTH-WEST UNIVERSITY
NOORDWES-UNIVERSITEIT
YUNIBESITI YA BOKONE-BOPHIRIMA

Private Bag X1290, Potchefstroom
South Africa 2520

Tel: 018 299-1111/2222
Fax: 018 299-4910
Web: http://www.nwu.ac.za

UNIT FOR ENVIRONMENTAL SCIENCES AND MANAGEMENT

Tel: 018 299 2372
Fax 018 299 1544
Email: Louis.duPreez@nwu.ac.za

DATE 13 July 2022

Dear Editor

Attached please find a manuscript titled "*Metapolystoma ohlerianum* n. sp. (Monogenea: Polystomatidae) from *Aglyptodactylus madagascariensis* (Anura: Mantellidae)" for possible inclusion in *Acta Parasitologica*.

The manuscript provides a description of a new *Metapolystoma* from Madagascar. We provide additional information on the genus and support the description with molecular evidence, histology and SEM. The paper furthermore address the validity of the genus *Metapolystoma*. We are currently finalizing a book on "Polystomes of the World" and this paper will contribute significantly to clarify the position of *Metapolystoma* as a valid genus.

<u>Compliance with ethical standards</u>

Ethical clearance was obtained from the North-West Uinivirsity Anim-Care Ethical committee (NWU-00380-16-A5-04).

<u>Conflict of interest</u>: None

<u>Author contribution:</u>

Conceptualization, Methodology, Formal analysis, Writing and review (Willem Landman, Olivier Verneau and Louis du Preez); Writing of original draft (Willem Landman); Funding acquisition (Louis du Preez); Supervision (Olivier Verneau and Louis du Preez).

<u>Plagerism</u>

The information provided in this manuscript is original and has not been published elsewhere, however, it formed part of the unpublished masters distertation of Willem Landman.

Kind regards

Willem Landman
